# Sustainable fabrication of 2D-based devices through reuse of substrates with microfabricated electrodes

**DOI:** 10.3762/bjnano.17.58

**Published:** 2026-06-18

**Authors:** Ying Zhang, Yigit Sozen, Esteban Zamora-Amo, Thomas Pucher, Nuria Jiménez-Arévalo, Zdenek Sofer, Yong Xie, Andres Castellanos-Gomez

**Affiliations:** 1 2D Foundry research group. Instituto de Ciencia de Materiales de Madrid (ICMM-CSIC), Madrid, 28049, Spain; 2 Universidad Autónoma de Madrid, Escuela de Doctorado, Madrid, 28049, Spain; 3 Department of Inorganic Chemistry, University of Chemistry and Technology, Prague, Prague, Czech Republic; 4 School of Advanced Materials and Nanotechnology, Xidian University, 710071 Xi’an, China

**Keywords:** 2D device fabrication, microfabricated electrode, *N*-methyl-2-pyrrolidone, substrate reuse, ultrasonic cleaning

## Abstract

Fabricating microelectronic devices for two-dimensional (2D) materials research is essential but often limited by the high cost and need for specialized facilities. This study establishes a practical method for cleaning and reusing substrates with pre-patterned electrodes. The cleaning protocol involves the use of an ultrasonic bath in warm *N*-methyl-2-pyrrolidone (NMP), enabling the removal of 2D materials without damaging the electrodes. Electrical measurements, Raman analysis, and Kelvin probe force microscopy measurements collectively confirm the feasibility of repeatedly reusing the same pre-patterned chip, showing that the cleaned regions exhibit no detectable Raman signatures of the transferred 2D material, retain a largely homogeneous surface-potential distribution, and preserve comparable electrical performance after reuse. By extending the lifetime of pre-patterned chips, this approach can reduce substrate consumption and lower the cost of 2D device prototyping.

## Introduction

Nanoscience research often needs the fabrication of proof-of-concept devices to demonstrate applications of novel nanomaterials or to study their fundamental properties [1–4]. Creating these microelectronic devices requires access to highly specialized infrastructure like cleanrooms and trained personnel [5–7]. Consequently, research groups focused on nanomaterials synthesis may lack the resources to integrate their novel nanomaterials into microelectronic devices, potentially reducing the impact of their research.

To address this issue, commercially available substrates with pre-patterned electrodes, ready for integration with the nanomaterial under study, offer a practical alternative [7–9]. In recent years, our team has regularly employed this strategy, transferring two-dimensional (2D) materials onto pre-patterned electrodes to create devices such as transistors, photodetectors, and diodes [10–15]. However, this approach incurs significant costs, approximately 40–50 € per chip for custom-made electrodes. Therefore, developing a reliable method for cleaning and reusing these devices would be valuable for achieving more cost-effective 2D material-based device fabrication.

Several recent studies have explored substrate reuse strategies to mitigate fabrication costs and improve sustainability in device prototyping [16–18]. For example, Bhalla et al. investigated various cleaning and regeneration techniques for electrochemical sensor chips, comparing piranha solution, plasma, oxidative, and reductive electrochemical cleaning methods [19]. Similarly, Stan et al. showed different cleaning methods for screen-printed gold electrodes and identified optimal techniques that allow for their reuse in electrochemical applications, preserving electrode performance [20]. Furthermore, Fakhr et al. explored cleaning methods for gold electrodes on diverse substrates and found that chemical treatments such as KOH and H_2_O_2_ effectively restore the substrate surface, allowing for their application in reusable biosensors [21]. A different approach to reuse the substrates was reported by Paupy et al. by developing wafer-scale detachable monocrystalline germanium nanomembranes for III–V material growth and substrate reuse, significantly reducing material waste and enabling repeated use of substrates in epitaxial growth applications [22].

Building on these previous studies, we propose a reliable and reproducible cleaning process for reusing microfabricated substrates with pre-patterned electrodes in 2D materials research. Previous studies have mainly focused on epitaxial growth or electrochemical sensing applications. In contrast, this work addresses the reuse of substrates for 2D material-based electronic devices. The cleaning process consists of an ultrasonic bath in *N*-methyl-2-pyrrolidone (NMP) [23–24] at 50 °C, followed by acetone and isopropyl alcohol (IPA) rinse and nitrogen blow-drying. This procedure is designed to remove residual 2D materials and adhesives from the substrates. The selection of cleaning solvent is critical and guided by the physical properties required to overcome the strong adhesion at the 2D material/substrate interface. In liquid-phase exfoliation (LPE) [25–26], polar aprotic solvents such as NMP and dimethylformamide (DMF) [27] have long been recognized as effective solvents for layered materials [28–29]. In this work, both NMP and DMF deliver strong cleaning performance under our post-transfer cleaning conditions. Dihydrolevoglucosenone (Cyrene) has recently emerged as a greener alternative within this class, with solubility parameters and surface tension similar to those of NMP and DMF, but a lower toxicity profile [30–31]. Overall, these observations suggest that the solvent classes effective for layered materials in LPE may also guide solvent selection for post-transfer cleaning. Accordingly, Cyrene represents an attractive greener candidate for future study, while NMP is adopted here as the representative solvent for systematic investigation of the cleaning protocol and substrate reuse.

This cleaning protocol enables substrates to be reused without pronounced degradation in device performance. By extending the usable lifetime of expensive microelectronic substrates, the approach reduces substrate consumption and provides a more cost-effective route for prototyping 2D material-based electronic devices, making the fabrication process more practical for laboratories with limited access to cleanroom or microfabrication facilities.

## Results and Discussion

**Figure 1:**
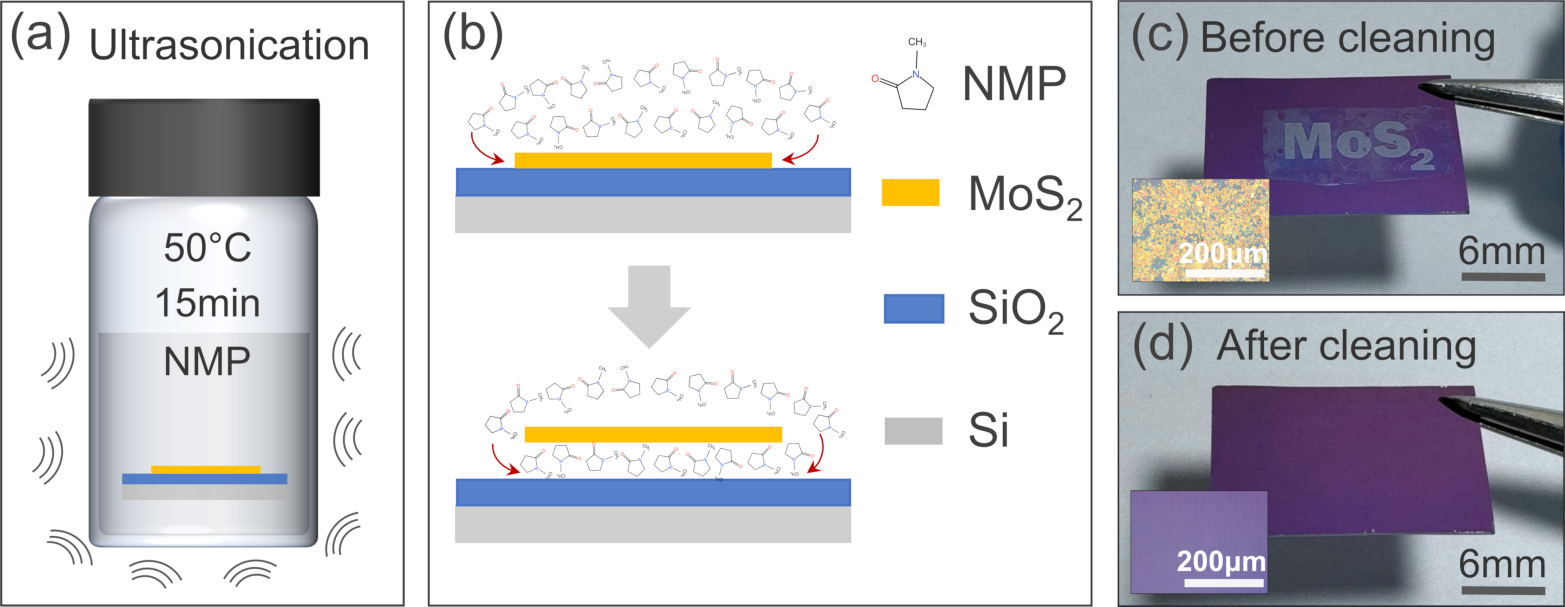
Demonstration of the ultrasonic cleaning process for substrate reuse. (a) Schematic diagram illustrating the ultrasonic cleaning setup with *N*-methyl-2-pyrrolidone (NMP) at 50 °C to remove MoS_2_ flakes and adhesive residues from the substrate. (b) Schematic representation illustrates the removal of MoS_2_ flakes from the substrate through the penetration of *N*-methyl-2-pyrrolidone (NMP) at 50 °C. (c, d) Optical images of the substrate before and after the removal of MoS_2_ flakes fabricated via roll-to-roll mechanical exfoliation.

Figure 1a presents a schematic illustration of the cleaning process: The chip with pre-patterned electrodes that needs to be cleaned is immersed in NMP and treated in an ultrasonic bath at 50 °C. The effectiveness of the ultrasonic cleaning is likely related to the interaction between NMP molecules and the interface between the 2D material flakes and the SiO_2_/Si substrate. NMP is known for its strong affinity for surface contaminants and its ability to penetrate microscopic gaps [24]. Heating to 50 °C can help weaken the van der Waals interactions between the 2D flakes and the substrate. Simultaneously, ultrasound agitation enhances molecular diffusion and promotes cavitation, generating localized pressure fluctuations that further assist in lifting the flakes. As a result, NMP molecules may penetrate the interface between the 2D material and the SiO_2_ surface, which assists flake detachment while preserving the visible integrity of the underlying electrodes (see schematic illustration in Figure 1b).

As an initial test of the cleaning protocol, we applied it to a SiO_2_/Si substrate covered with a continuous MoS_2_ flakes network prepared by five successive transfers via high-throughput roll-to-roll mechanical exfoliation [32–33]. This method enables a high surface coverage of densely packed flakes that adhere strongly to the substrate, making it a more challenging and representative benchmark for cleaning compared to conventional manual exfoliation, which typically results in sparsely distributed flakes that are easier to remove. The MoS_2_ film was patterned using a vinyl stencil mask, producing a well-defined ‘MoS_2_’ pattern on the substrate surface. As shown in Figure 1c, prior to cleaning, the substrate exhibited both the patterned MoS_2_ network and residual adhesive from the stencil mask around the ‘MoS_2_’ area. Following the ultrasonic bath in hot NMP and subsequent rinsing in isopropanol, all MoS_2_ flakes and visible adhesive residues were removed from the patterned region, leaving a pristine SiO_2_/Si surface (Figure 1d). The insets in Figure 1c and Figure 1d provide high-magnification optical microscopy images. Before cleaning, the interconnected network of MoS_2_ flakes is visible, whereas after treatment, the surface appears clean and free of contaminants.

The rationale for selecting NMP as the representative cleaning solvent and 50 °C as the operating temperature is discussed in detail in Supporting Information File 1 (see Figures S1–S4), where the performance of alternative solvents under representative cleaning conditions is also compared. The similarly high cleaning efficiency observed for both NMP and DMF, even for samples stored for over six months (see Supporting Information File 1, Figure S2), indicates that effective cleaning can be achieved by more than one highly polar aprotic solvent under the present conditions. As summarized in Table S1 (Supporting Information File 1), NMP and DMF possess surface tensions (γ) and dispersive solubility parameters (δ_D_) that closely match the surface energy characteristics of MoS_2_ (γ ≈ 40 mN/m, δ_D_ ≈ 18 MPa^1/2^), whereas dimethyl sulfoxide (DMSO) and acetone deviate more strongly from this optimal range and therefore perform less effectively, especially for aged flakes. These observations suggest that solvent classes known to perform well for layered materials may also provide a useful basis for selecting cleaning solvents for post-transfer substrate reuse. Based on this consideration, Cyrene is an especially interesting greener candidate, although it was not experimentally evaluated as a cleaning solvent in the present study. As a representative solvent, NMP is used in the main manuscript for the systematic study.

**Figure 2:**
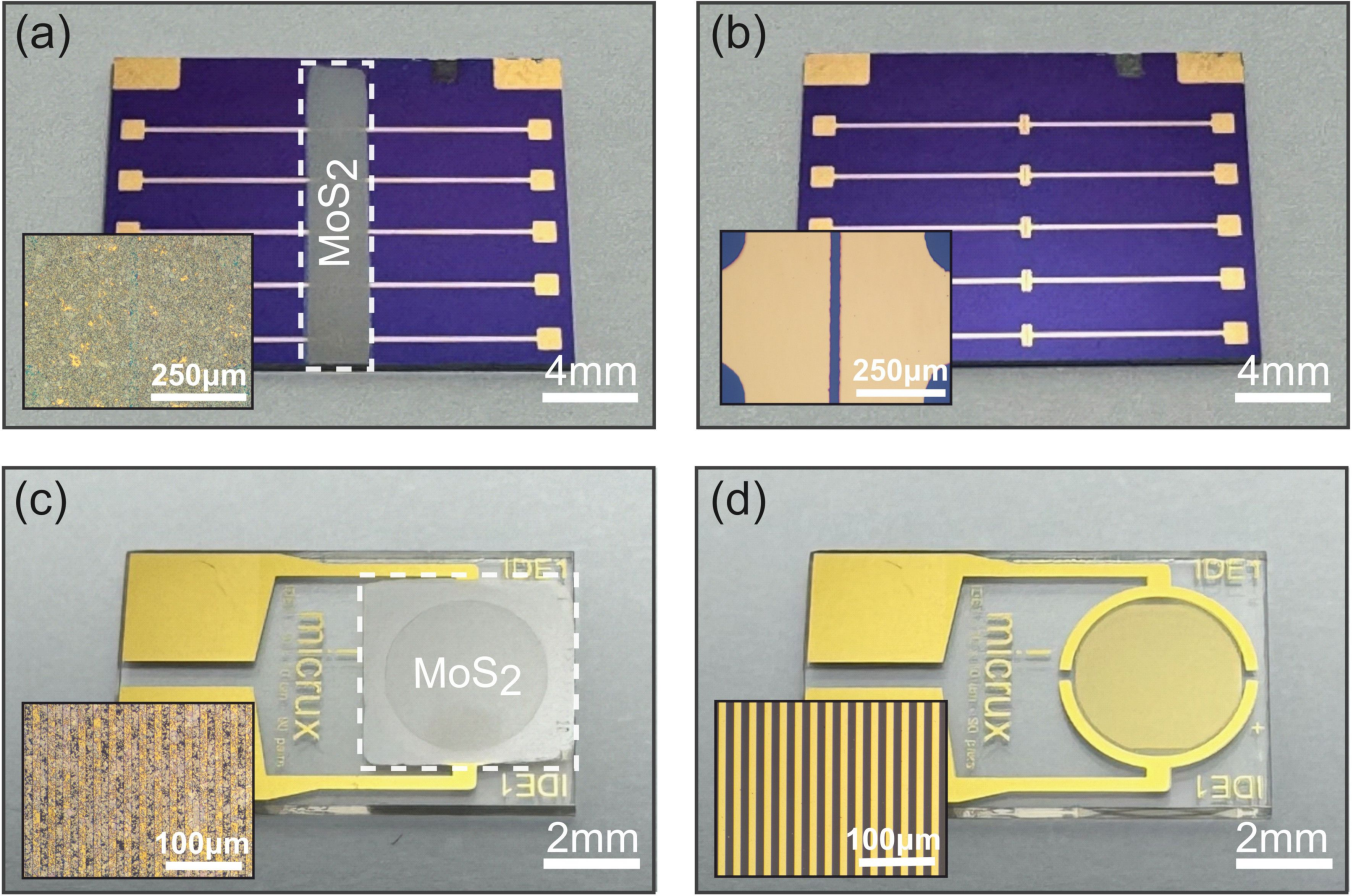
Substrates with different pre-patterned electrodes before and after cleaning. (a, b) Macroscopic images of a SiO_2_/Si substrate with five pre-patterned Cr/Au drain-source electrodes (channel length: 25 µm). A large amount of MoS_2_ flakes was transferred onto the electrode regions prior to the cleaning procedure, as shown in (a). Images were taken before (a) and after (b) the cleaning process. Insets show representative optical microscopy images for each case. (c, d) Macroscopic images of a Ti/Au interdigitated electrode on glass (Micrux) with a channel length of 10 µm. MoS_2_ films had been transferred onto the electrode regions prior to cleaning, as shown in (c). Images were taken before (c) and after (d) the cleaning process. Insets show representative optical microscopy images for each case. The dashed boxes outline the MoS_2_ film region.

The cleaning process was also applied to substrates with different electrode layouts and metal stacks. Figure 2 presents different pre-patterned electrode configurations before and after the cleaning process. Figure 2a,b shows drain–source electrode structures pre-patterned on a SiO_2_/Si substrate using a Cr/Au stack, commonly used in field-effect transistor (FET) fabrication, initially covered with a large-scale MoS_2_ film produced by roll-to-roll mechanical exfoliation. Figure 2c,d displays a Ti/Au interdigitated electrode on glass with the 10 µm gap separation, widely used for electrochemistry and biosensing applications [34]. For both cases, the cleaning process effectively removes the MoS_2_ flakes while preserving the structural integrity of the electrodes, indicating that the method is compatible with different adhesion layers used beneath Au. The insets in Figure 2 provide zoomed-in optical images that further illustrate the removal of material from the electrode gaps. In addition to the cleaning of films of roll-to-roll mechanically exfoliated van der Waals materials, we investigated the cleaning and reuse of substrates with chemical vapor deposition (CVD)-grown MoS_2_ flakes (see Supporting Information File 1, Figure S5) [35–36].

**Figure 3:**
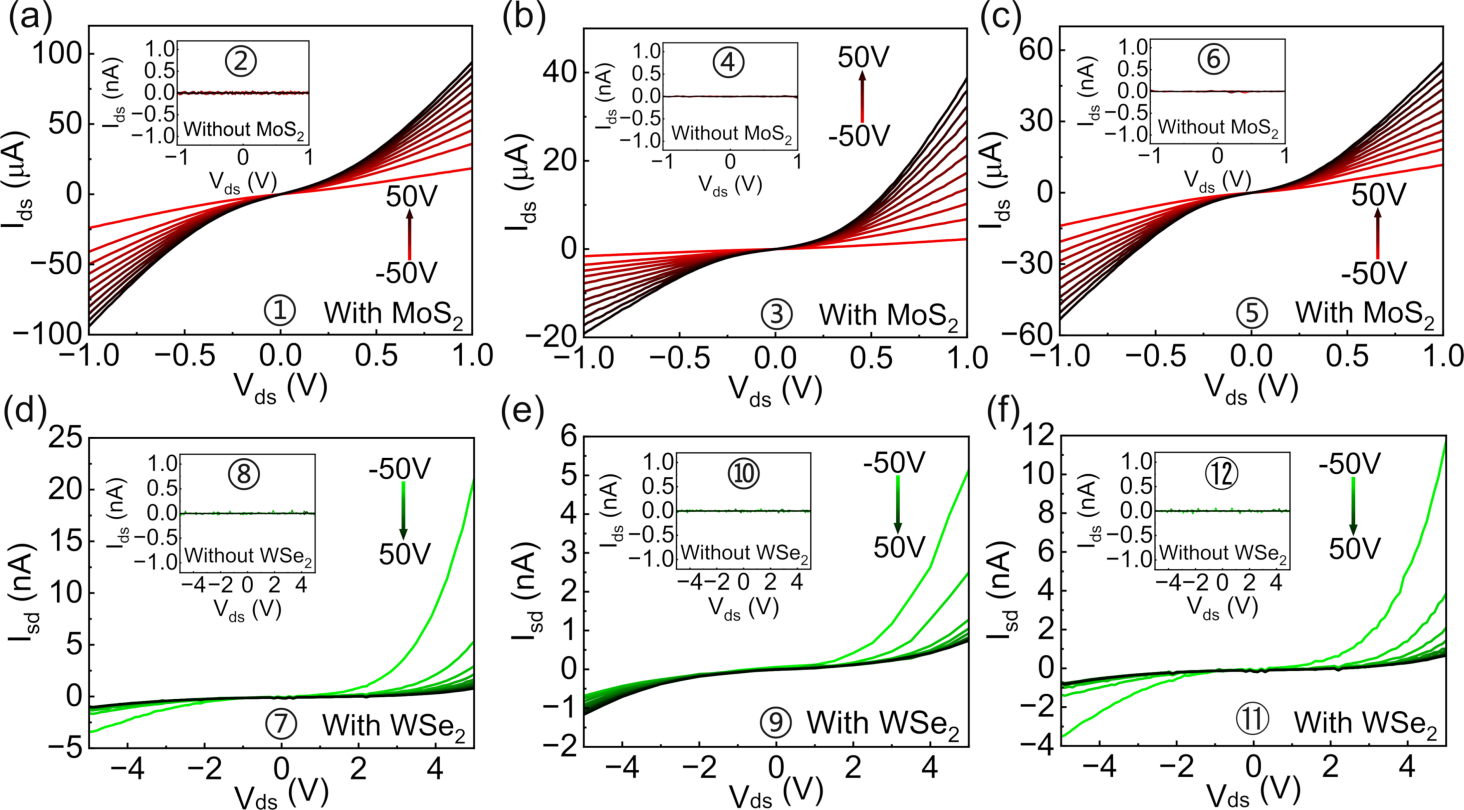
Repeated characterization of MoS_2_- and WSe_2_-based FETs on the same pre-patterned SiO_2_/Si chip before and after multiple cleaning processes. (a–c) Drain–source current versus bias voltage characteristics of the devices at different gate voltages of the first, second, and third MoS_2_-based FETs. (d–f) Drain–source current versus bias voltage characteristics of the devices at different gate voltages of the first, second and third WSe_2_-based FETs, following three successful MoS_2_-based FETs. The insets in each plot present the drain-source current versus bias voltage characteristics of the same sample measured after each successive cleaning step.

To examine the feasibility of repeated substrate reuse, we performed multiple cycles of device fabrication, electrical characterization, and subsequent cleaning on a microfabricated SiO_2_/Si chip with Ti/Au electrodes. MoS_2_ flakes obtained via roll-to-roll mechanical exfoliation were sequentially transferred onto the same pre-patterned electrodes to fabricate three FETs [32,37]. After each fabrication, the drain–source current versus bias voltage (*I*–*V*) characteristics were measured at different gate voltages, as shown in Figure 3a–c. Following the electrical characterization, the samples underwent the cleaning process, and the substrates were tested to verify the absence of any electrical connections between the electrodes. The insets in Figure 3a–c show the electrical characterization after each cleaning process, confirming the absence of residual conductivity between the electrodes, indicating effective removal of the conductive channel material from the active device region.

Although some variations were observed in the electrical characteristics of the re-fabricated FETs, this is expected due to the inherent stochastic nature of the MoS_2_ films fabricated by roll-to-roll mechanical exfoliation, which consist of a network of interconnected flakes [32]. Nonetheless, the overall reproducibility of device operation across repeated fabrication cycles demonstrates that the cleaning process preserves the functionality of the pre-patterned electrodes and enables effective reuse of the substrates.

To assess the applicability of this cleaning method to other 2D materials, we extended the study to WSe_2_-based devices. After three successful cycles with MoS_2_, we transferred WSe_2_ flakes onto the same cleaned electrodes and fabricated three additional FETs following the same procedure. Representative macroscopic images of these devices are shown in Supporting Information File 1, Figure S6. The electrical characteristics of these devices, measured before and after each cleaning cycle, are presented in Figure 3d–f. The measurements indicate that the cleaning method can be applied to both MoS_2_ and WSe_2_ devices without causing noticeable degradation of the electrode performance. Overall, the same pre-patterned SiO_2_/Si chip can be reliably reused for at least six consecutive fabrication/cleaning cycles, while maintaining electrical isolation after each cleaning step and preserving the functionality of the electrodes for subsequent device fabrication. These results show that the cleaning process can support repeated substrate reuse under the tested conditions and reduce the need for newly fabricated chips.

The cleaning process was also applied to MoS_2_ and WSe_2_ films on indium tin oxide (ITO) substrates, as shown in Supporting Information File 1, Figure S7. The fabrication and cleaning procedures were carried out as previously described in this work. As shown in Supporting Information File 1, Figure S8, the electrical characterization of the devices before and after cleaning confirms the highly effective removal of MoS_2_ and WSe_2_ flakes, with no visible residues. Additionally, the ITO electrodes did not show clear electrical degradation after repeated use, indicating that the process is also compatible with this electrode platform.

**Figure 4:**
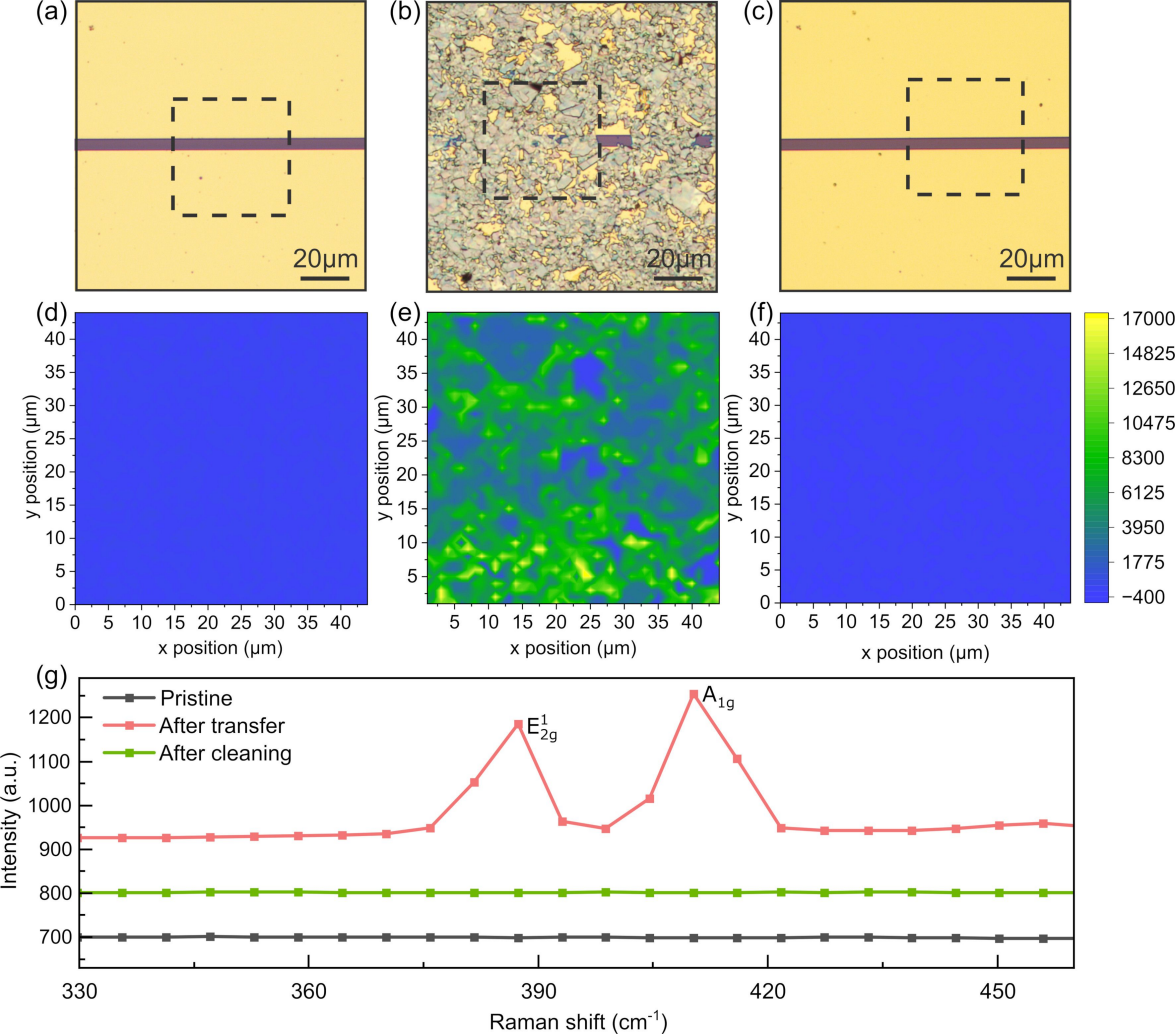
Raman analysis of the electrode surface in the pristine state, after roll-to-roll mechanically exfoliated MoS_2_ transfer, and after NMP cleaning. (a–c) Optical microscopy images of the electrode region in the pristine state, after MoS_2_ transfer, and after NMP cleaning, respectively. The dashed outlines indicate the areas selected for Raman mapping. (d–f) Processed interpolated Raman integrated-intensity maps of the corresponding mapped regions, obtained by integrating the MoS_2_ characteristic spectral window (370–425 cm^−1^) after linear baseline correction. (g) Average Raman spectra extracted from the mapped areas for the three states. The corresponding raw pixelated Raman maps are provided in Supporting Information File 1.

Raman mapping of the active region was performed to further assess the cleanliness of the electrode surface in three states, namely, pristine electrode, electrode after roll-to-roll mechanically exfoliated MoS_2_ transfer, and electrode after NMP cleaning. Figure 4a–c shows optical microscopy images of the corresponding regions, with the dashed outlines indicating the areas selected for Raman mapping. For a quantitative comparison, each Raman spectrum collected from the mapping pixels was first corrected with a linear baseline, and the MoS_2_-related signal was then evaluated by integrating the intensity in the 370–425 cm^−1^ spectral range. This window includes the main Raman modes of MoS_2_ and provides a more stable measure than a single-peak intensity, particularly when the signal is weak or the peak position changes slightly.

The resulting processed interpolated Raman maps are shown in Figure 4d–f. For the pristine electrode, the integrated Raman intensity remains at the background level. After MoS_2_ transfer, a clear Raman signal is observed over the mapped region, confirming the presence of transferred material on the electrode surface. After NMP cleaning, the Raman signal decreases to a level comparable to that of the pristine state. The average Raman spectra extracted from the mapped areas, shown in Figure 4g, display the same trend, with clear MoS_2_-related features observed after transfer and the signal suppressed to the background level after cleaning.

These results suggest that, within the detection limit of the Raman mapping used here, no clear MoS_2_ residue remains in the analyzed active region after NMP cleaning. This observation provides spectroscopic evidence that is consistent with the optical and electrical results. The raw pixelated Raman maps in Supporting Information File 1, Figure S9d–f show the same change, confirming that the observed contrast is not introduced by interpolation. The Si 520 cm^−1^ maps in Supporting Information File 1, Figure S9g–i provide additional contrast between the SiO_2_/Si channel and the Au electrode.

In addition, Kelvin probe force microscopy (KPFM) measurements, presented in Supporting Information File 1 (Figures S10 and S11), show that the electrode surface after MoS_2_ transfer followed by NMP cleaning retains a mostly homogeneous surface-potential distribution. Although the cleaned electrode exhibits a slightly broader surface-potential histogram than the pristine one, no clear large-area electrostatic inhomogeneity is observed, suggesting that the cleaning process largely preserves the surface-potential uniformity of the electrode.

**Figure 5:**
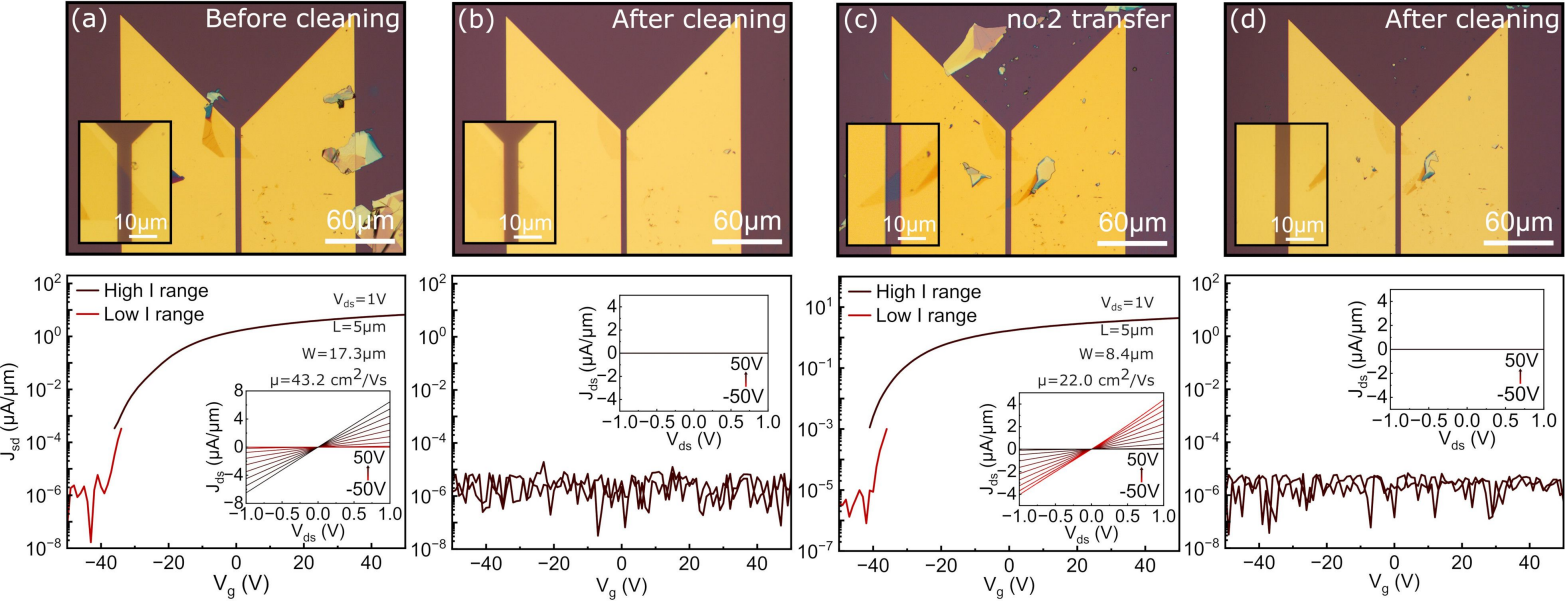
Reproducible fabrication, cleaning, and characterization of monolayer MoS_2_ FETs on the same pre-patterned chip. (a, b) Microscopic images of the first monolayer MoS_2_ flake bridging the source–drain electrodes before and after cleaning, together with the transfer characteristics and drain–source current versus bias voltage characteristics at different gate voltages. (c, d) Microscopic images and electrical characteristics of a second monolayer MoS_2_ flake transferred onto the same electrodes before and after cleaning. Insets in each panel show magnified macroscopic images of the monolayer MoS_2_ region before and after cleaning.

Beyond the roll-to-roll deposited films of 2D materials, it is crucial to evaluate the reusability of substrates in the context of devices fabricated with single mechanically exfoliated flakes or CVD-grown films (approaches more commonly adopted in the literature). To this end, we demonstrate the recycling of a pre-patterned electrode substrate for fabricating single-flake MoS_2_ field-effect transistors (FETs). Single-layer MoS_2_ flakes were identified using transmission-mode optical microscopy and confirmed via differential reflectance spectroscopy [38–40]. A selected flake was then transferred using an all-dry deterministic method to bridge a pair of gold electrodes (Figure 5a, top) [41]. After high-vacuum annealing, the device was electrically characterized in a custom high-vacuum probe station [42]. Figure 5a (bottom) presents representative output and transfer characteristics of the single-layer MoS_2_ FET, displaying an on/off current ratio of 3.4 × 10^6^ and a field-effect mobility of 43.2 cm^2^·V^−1^·s^−1^.

Following characterization, the device underwent the deep cleaning protocol. The process effectively removed the MoS_2_ from the channel region and detached most multilayer residues. However, small remnants of the monolayer in direct contact with the gold electrodes remained (see inset in Figure 5b), likely due to the strong chemical affinity between MoS_2_ and gold (a mechanism exploited in gold-assisted exfoliation methods) [43–44]. Interestingly, in devices that had not been annealed, the entire flake was more easily removed, presumably due to weaker adhesion resulting from interfacial adsorbates or trapped air (see Supporting Information File 1, Figure S12).

Despite the incomplete removal of the monolayer at the gold contacts, the electrodes remained electrically isolated after cleaning, as confirmed by the open-circuit behavior shown in Figure 5b. A new flake was then transferred a few micrometers away, forming a second FET that exhibited typical device performance (Figure 5c), with an on/off current ratio of 8.5 × 10^5^ and a field-effect mobility of 22.0 cm^2^·V^−1^·s^−1^. Finally, the device underwent an additional cleaning cycle, restoring the substrate for further reuse (Figure 5d), thus completing the full reusability cycle.

## Conclusion

The reuse of microelectronic substrates with pre-patterned electrodes can reduce fabrication costs and improve the use of laboratory resources. In this work, we developed and validated a reliable cleaning strategy for the reuse of microelectronic substrates with pre-patterned electrodes, based on ultrasonic treatment in NMP followed by rinsing in acetone and IPA and nitrogen drying. Electrical measurements show that the same pre-patterned SiO_2_/Si chip could be reused for at least six fabrication/cleaning cycles while preserving electrical isolation after each cleaning step and maintaining electrode functionality for subsequent device fabrication.

The cleaning effect is further supported by Raman mapping, which shows that the MoS_2_-related Raman signal is suppressed to the background level after cleaning. Furthermore, KPFM measurements indicate that the cleaned electrode retains a largely homogeneous surface-potential distribution, suggesting that the cleaning process does not introduce pronounced electrostatic inhomogeneity. In addition to MoS_2_-based devices, the method is also applicable to WSe_2_ and ITO-based platforms and can be extended to CVD-grown films and manually exfoliated monolayer flakes.

Although the main manuscript adopts NMP as the representative cleaning solvent, Supporting Information File 1 shows that DMF exhibits comparable cleaning efficiency under the tested conditions, whereas DMSO and acetone are less effective. These observations suggest that solvents known to perform well for layered materials can also help guide solvent selection for post-transfer cleaning. In this context, Cyrene is an attractive greener candidate for future study, although it was not experimentally evaluated here.

Overall, the results support substrate reuse as a practical strategy to reduce substrate consumption and promote more sustainable use of resources in 2D materials device fabrication. Future work could explore this cleaning protocol with other material systems and electrode architectures, as well as further quantify the long-term durability of reused substrates across multiple cycles.

## Experimental

### Pre-patterned electrodes fabrication

Pre-patterned electrodes used in this work were obtained from different sources. The SiO_2_ (290 nm)/Si (p++) substrate shown in Figure 2a,b was prepared by placing a shadow mask (Ossila E291) onto the substrate, followed by thermal evaporation of 5 nm Cr/45 nm Au. All other Au-based electrodes used in this work employed a 5 nm Ti/45 nm Au stack deposited by electron-beam evaporation. The interdigitated electrodes on glass were supplied from Micrux Technologies and also employed a 50 nm Ti/150 nm Au stack. The interdigitated ITO electrodes on glass were purchased from Ossila (S161-20).

### Materials preparation

The MoS_2_-based samples were prepared using natural bulk molybdenite mineral (Molly Hill Mine, Quebec, Canada). WSe_2_ crystals were prepared by CVT method from tungsten and selenium using bromine as a transport agent. High-throughput mechanical exfoliation of large-scale MoS_2_ and WSe_2_ was carried out using a roll-to-roll setup. Nitto SPV 224 tape was applied on the surfaces of two polyoxymethylene cylinders with a perimeter ratio of 53:23 [32]. A bulk van der Waals material was placed on one cylinder, and the system was rotated under moderate pressure (20–40 N) at ≈1500 rpm for 50 s, resulting in uniformly exfoliated large-area flakes adhered to the tape surface. Monolayer MoS_2_ flakes were obtained by mechanical exfoliation of bulk MoS_2_ crystals using Nitto SPV 224 tape and subsequently transferring the exfoliated flakes onto Gel-Film (WF 4× 6.0 mil). Candidate monolayer regions were first identified by transmission optical microscopy and then confirmed by differential reflectance spectroscopy [38–40]. Monolayer CVD-MoS_2_ flakes were grown by chemical vapor deposition using a NaCl-assisted ambient-pressure CVD approach, following established procedures reported in the literature [35–36]. After growth, the samples were cleaned using the same protocol applied to mechanically exfoliated flakes.

### Transfer process

Transfer of large-area high-throughput flakes was carried out by bringing the tape containing the exfoliated flakes into conformal contact with the target substrate, followed by gentle pressing using a cotton swab to promote adhesion. The sample was then annealed on a hotplate at 110 °C for 5 min to facilitate the transfer of films composed of MoS_2_ or WSe_2_ flakes. To ensure high-density coverage of the flakes, multiple sequential transfer steps were employed. For electronic device fabrication, this transfer process was typically repeated three to five times. Mechanically exfoliated (manual) monolayer MoS_2_ flakes were transferred onto the pre-patterned electrodes using the deterministic dry-transfer technique [41].

### Electrical characterization

The drain–source current versus bias voltage and FET characteristics were measured using a custom-built probe station and a Keithley 2450 source-meter unit. Additionally, two programmable benchtop power supplies (TENMA, model 72-2715) were connected in series to characterize the FETs output at varying back-gate voltages from −50 V to 50 V. Devices that underwent annealing were characterized under a vacuum of 1.5 × 10^−5^ mbar [42].

### Raman characterization

Raman mapping measurements were performed on the electrode region in three states, namely, pristine electrode, electrode after roll-to-roll mechanically exfoliated MoS_2_ transfer, and electrode after NMP cleaning. The mapping data were exported as individual spectra at each mapped pixel. For each spectrum, the Raman spectrum was linearly baseline-corrected using background regions outside the MoS_2_ characteristic window, and the MoS_2_-related signal was quantified by integrating the 370–425 cm^−1^ spectral range. This region includes the main MoS_2_ Raman modes and was used to construct the Raman integrated-intensity maps shown in the main text and Supporting Information File 1. Average Raman spectra were obtained by averaging all spectra within the mapped region for each state.

### Kelvin probe force microscopy

KPFM measurements were carried out on the same electrode in two states, that is, pristine electrode and electrode after roll-to-roll mechanically exfoliated MoS_2_ transfer followed by NMP cleaning. Morphology and surface-potential characterization were performed under ambient conditions using a commercial AFM system (NanoObserver, CSI Instruments). Topographic images were acquired in dynamic mode using the oscillation amplitude as the feedback signal. Surface-potential maps were recorded in single-pass KPFM mode (HD-KFM) using an Au-coated probe (PPP-FMAu, Nanosensors), with an AC voltage of 1.5 V applied to the tip. Both large-area and higher-magnification scans were collected to compare the topography and surface-potential (SP) distribution before and after processing. SP histograms were extracted from the mapped regions to compare the width of the surface-potential distribution. All image analysis was carried out using the open-source Gwyddion software.

### Cleaning process

The fabricated FET devices were immersed in 10 mL of *N*-methyl-2-pyrrolidone (Sigma-Aldrich) and ultrasonicated at 50 °C for 15 min or in repeated cycles of 15 min using an ultrasonic cleaner (from RS PRO). Unless otherwise stated, *N*-methyl-2-pyrrolidone (Sigma-Aldrich) was used as the representative cleaning solvent in the main study. After ultrasonic treatment, the devices were rinsed sequentially with acetone and isopropyl alcohol, and dried with nitrogen. Comparative cleaning tests were additionally performed using dimethylformamide, dimethyl sulfoxide (TechniStrip Micro D350), and acetone under analogous conditions, as discussed in Supporting Information File 1.

## Supporting Information

Supporting Information includes additional figures, experimental procedures, and supplementary data that support the findings presented in the main text.

File 1 Supplementary figures and experimental details.

## Data Availability

Data generated and analyzed during this study is openly available in Zenodo at https://doi.org/10.5281/zenodo.15835491.
