# Supplementary material for: Sustainable fabrication of 2D-based devices through reuse of substrates with microfabricated electrodes
Source: Beilstein J Nanotechnol. 2026 Jun 18;17:818–27. doi: 10.3762/bjnano.17.58 (PMC13284748; doi:10.3762/bjnano.17.58)
Supplement: File 1 — Supplementary figures and experimental details. [file Beilstein_J_Nanotechnol-17-818-s001.pdf]

BEILSTEIN JOURNAL OF NANOTECHNOLOGY

Supporting Information

for

Sustainable fabrication of 2D-based devices through reuse of substrates with microfabricated electrodes

Ying Zhang, Yigit Sozen, Esteban Zamora-Amo, Thomas Pucher, Nuria Jiménez-Arévalo, Zdenek Sofer, Yong Xie and Andres Castellanos-Gomez

Beilstein J. Nanotechnol. **2026,** *17,* 818–827. doi:10.3762/bjnano.17.58

Supplementary figures and experimental details

License and Terms: This is a supporting information file under the terms of the Creative Commons Attribution License (https://creativecommons.org/licenses/by/4.0). Please note that the reuse, redistribution and reproduction in particular requires that the author(s) and source are credited and that individual graphics may be subject to special legal provisions.
The license is subject to the *Beilstein Journal of Nanotechnology* terms and conditions: (https://www.beilstein-journals.org/bjnano/terms)

The mechanistic exploration was conducted under the optimized cleaning condition of ultrasonic treatment at 50 °C. To evaluate the solvent dependence and establish the standard cleaning protocol used in this work, we used MoS_2 films on SiO_2/Si prepared by high-throughput mechanical exfoliation as a model system and systematically compared the cleaning performance of four commonly used organic solvents - NMP, dimethylformamide (DMF), dimethyl sulfoxide (DMSO), and acetone (ACE) - at different temperatures (Figure S1). All cleaning experiments were performed approximately 24 h after transferring the material onto the substrates. For both NMP and DMF, the cleaning efficiency improved with increasing temperature, and at 50 °C a 15 min ultrasonic treatment was sufficient to remove the MoS_2 film to a nearly residue-free level by optical inspection. In contrast, although the cleaning efficiency of DMSO also improved markedly with increasing temperature, a small amount of residue remained at 50 °C. ACE showed the weakest performance overall, with substantial MoS_2 residues still visible on the substrate surface. These results indicate that, under our cleaning conditions, NMP and DMF provide similarly high cleaning efficiency, whereas DMSO and ACE are clearly less effective. In combination with the physicochemical comparison summarized in Table S1, these observations suggest that solvent classes known to perform well for layered materials may also provide useful guidance for post-transfer cleaning.

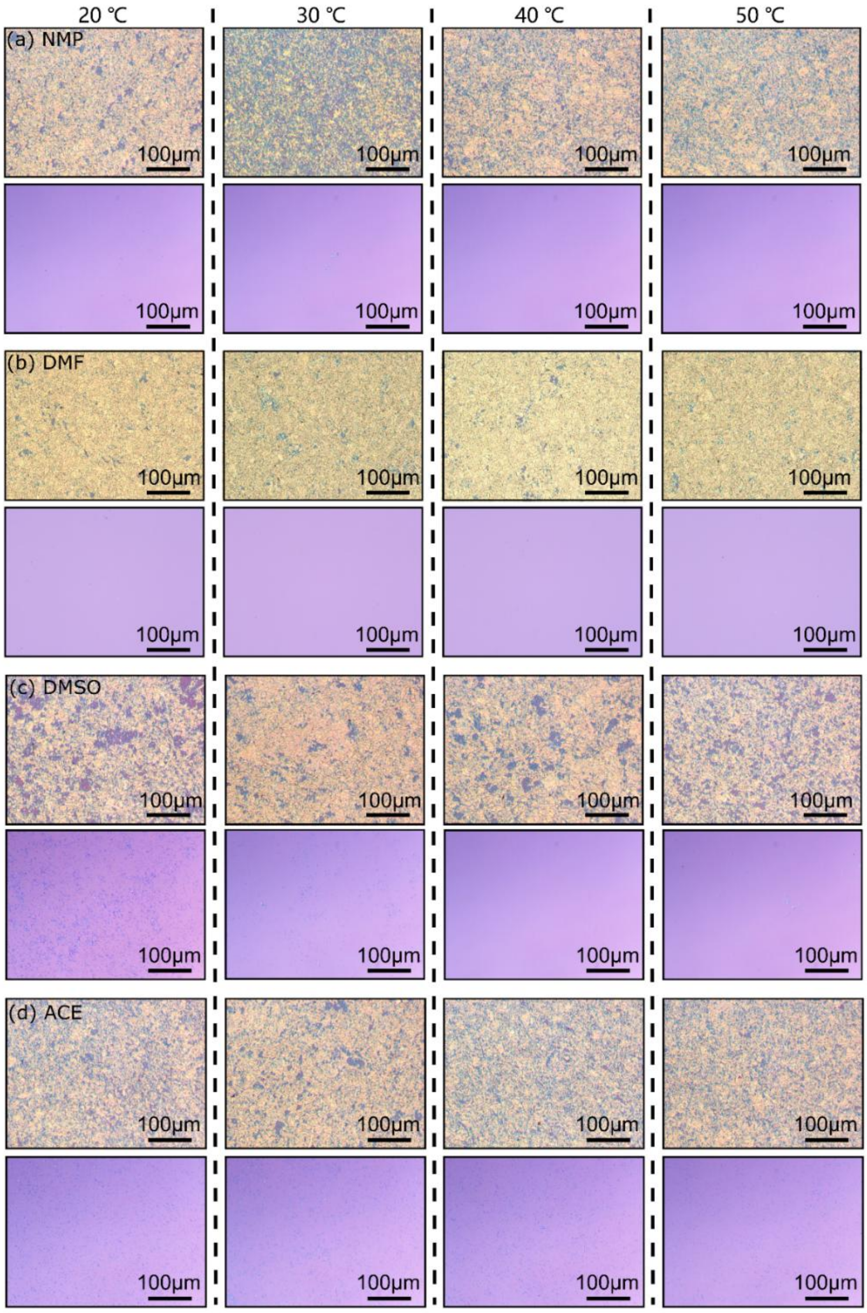

Figure S1: Temperature-dependent cleaning performance of different organic solvents on high-coverage MoS_2 network films. Optical micrographs of high-coverage MoS_2 network films on SiO_2/Si substrates prepared by high-throughput mechanical exfoliation, before and after ultrasonic cleaning at different temperatures using (a) NMP, (b) dimethylformamide (DMF), (c) dimethyl sulfoxide (DMSO), and (d) acetone (ACE), illustrating the solvent- and temperature-dependent evolution of the film morphology.

Table S1: Physical properties of selected organic solvents and their correspondence with the cleaning requirements for 2D material substrates.

Solvent	γ (mN/m)	δ_D ($MPa^{1/2}$)	δ_P ($MPa^{1/2}$)	δ_H ($MPa^{1/2}$)	η (mPa·s)
NMP	40.1	18.0	12.3	7.2	1.7
DMF	37.1	17.4	13.7	11.3	0.8
DMSO	43.5	18.4	16.4	10.2	2.0
ACE	23.3	15.5	10.4	7.0	0.3
Cyrene	44.4	18.8	10.6	6.9	14.5
Ideal range	40 ± 5	≈18	10-15	<10	--

Notably, Cyrene also lies close to the same physicochemical window defined by surface tension and Hansen solubility parameters. Together with the strong cleaning performance observed for NMP and DMF, this provides a useful rationale for considering Cyrene as a greener candidate for future post-transfer cleaning studies, although it was not experimentally tested here.

To further evaluate the robustness of the solvent choice under more challenging conditions, we took a substrate that had remained uncleaned for more than six months after MoS_2 transfer and divided the contaminated region into four comparable areas, which were then cleaned separately using NMP, DMF, DMSO, and ACE. The corresponding before/after optical images are shown in Figure S2. Under these conditions, both NMP and DMF were still able to remove the transferred material effectively, whereas DMSO and ACE left substantial visible residues. This comparison further supports the conclusion that NMP and DMF are the most effective solvents among those tested for the present post-transfer cleaning problem, including for long-stored samples. Together with their well-established role in LPE, these results suggest that this solvent class provides a useful starting point for identifying additional cleaning solvents for layered-material device reuse.

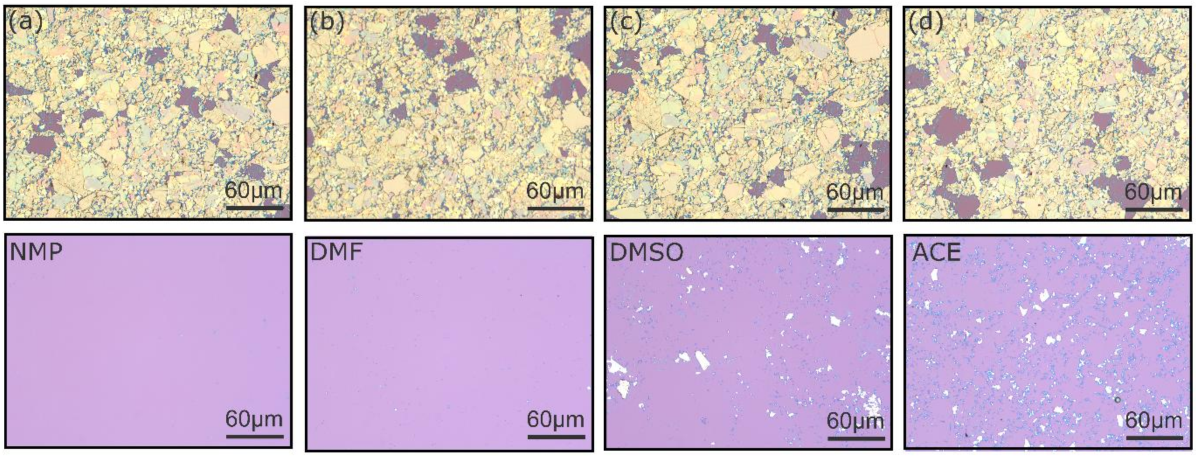

Figure S2: Solvent comparison for cleaning a long-stored transferred MoS_2 sample. Optical microscope images of a substrate that remained uncleaned for more than six months after MoS_2 transfer, with the contaminated region divided into four comparable areas and cleaned separately using NMP, DMF, DMSO, and ACE. The before/after comparison shows that NMP and DMF effectively remove the transferred material, whereas DMSO and ACE leave substantial visible residues.

In subsequent experiments, we further evaluated the behaviour of these solvents under more practical conditions by performing two consecutive MoS_2 transfer-cleaning cycles on the same pair of electrodes, in order to probe the effect of cross-usage and residence time. The first cleaning step was carried out on the day of MoS_2 transfer, while the second after a second transfer followed by three days of storage. The corresponding optical comparisons before and after cleaning are shown in Figure S3 (DMSO as the initial solvent) and Figure S4 (ACE as the initial solvent). When MoS_2 was cleaned on the day of transfer ("fresh" flakes), both DMSO and ACE removed the film reasonably well, leaving only minor residues. However, cross-cleaning did not provide any noticeable improvement in removing the remaining MoS_2 (ACE after DMSO and DMSO after ACE). For samples left on the electrodes for three days, the cleaning efficiency of both DMSO and ACE decreased significantly, and even an additional cleaning step in NMP could not achieve complete removal of the residual MoS_2, indicating that DMSO and ACE are markedly less effective for "non-fresh" flakes. In contrast, the high-coverage MoS_2 film shown in Figure 1c was cleaned directly using NMP after the sample had been left for a certain period, and a nearly residue-free surface was still obtained, demonstrating that NMP maintains high cleaning efficiency regardless of residence time. Therefore, despite their partial effectiveness, the cleaning capability of DMSO and ACE is overall inferior to that of NMP. Based on these results, unless otherwise specified, all subsequent experiments employ ultrasonic cleaning in NMP at 50 °C as the standard protocol.

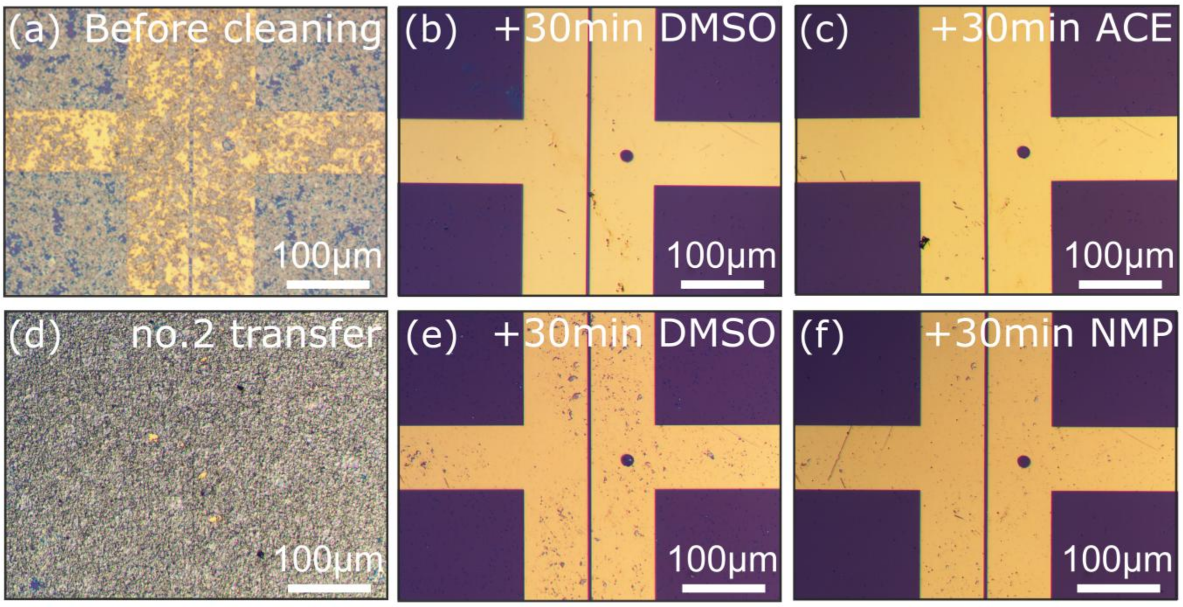

Figure S3: Optical microscope images comparing the electrode surface before and after ultrasonic cleaning with dimethyl sulfoxide (DMSO) on an electrode with transferred MoS_2. (a) Electrode image before cleaning after the first MoS_2 transfer; channel width is 5 μm. (b) Electrode image after 30 minutes of ultrasonic cleaning with DMSO on the day of transfer. (c) Electrode image following an additional 30-minute ultrasonic cleaning with ACE. (d) Electrode image before cleaning after the second MoS_2 transfer. (e) Electrode image after 30 minutes of ultrasonic cleaning with DMSO performed 3 days post-transfer. (f) Electrode image after a subsequent 30-minute ultrasonic cleaning with NMP. Elongated scratches visible in the images are attributed to the ultrasonic cleaning process.

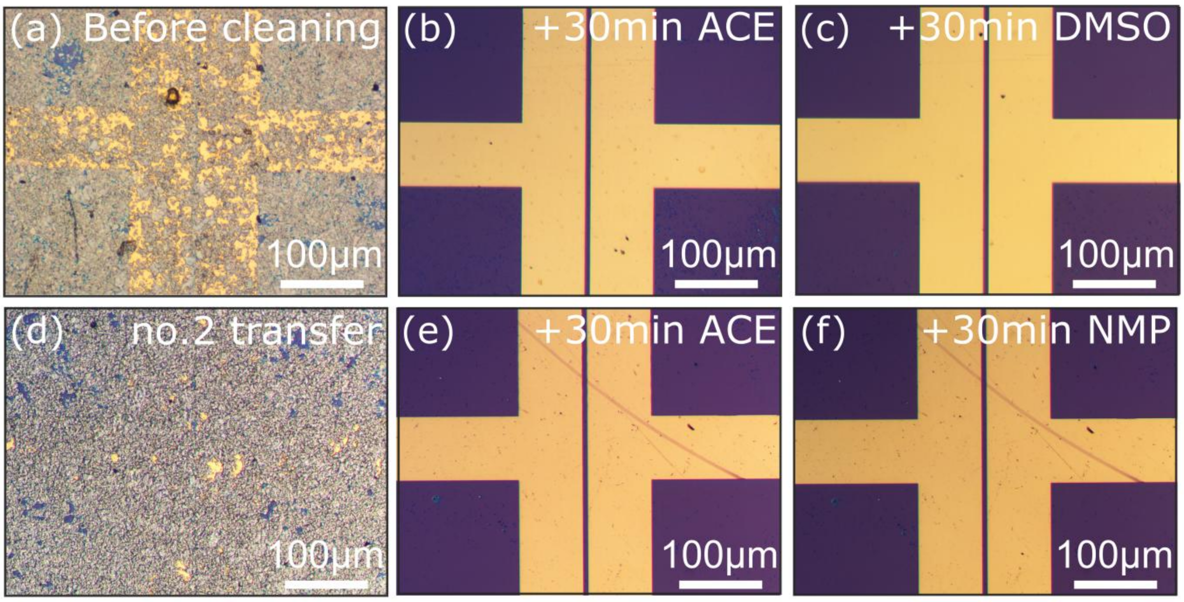

Figure S4: Optical microscope images showing the electrode surface before and after ultrasonic cleaning with ACE on an electrode with transferred MoS_2. (a) Electrode image before cleaning following the first MoS_2 transfer; channel width is 7 μm. (b) Electrode image after 30 minutes of ultrasonic cleaning with ACE on the day of transfer. (c) Electrode image after an additional 30 minutes of ultrasonic cleaning with DMSO. (d) Electrode image before cleaning following the second MoS_2 transfer. (e) Electrode image after 30 minutes of ultrasonic cleaning with ACE performed 3 days post-transfer. (f) Electrode image after a subsequent 30 minutes of ultrasonic cleaning with NMP. Elongated scratches visible in the images are attributed to the ultrasonic cleaning process.

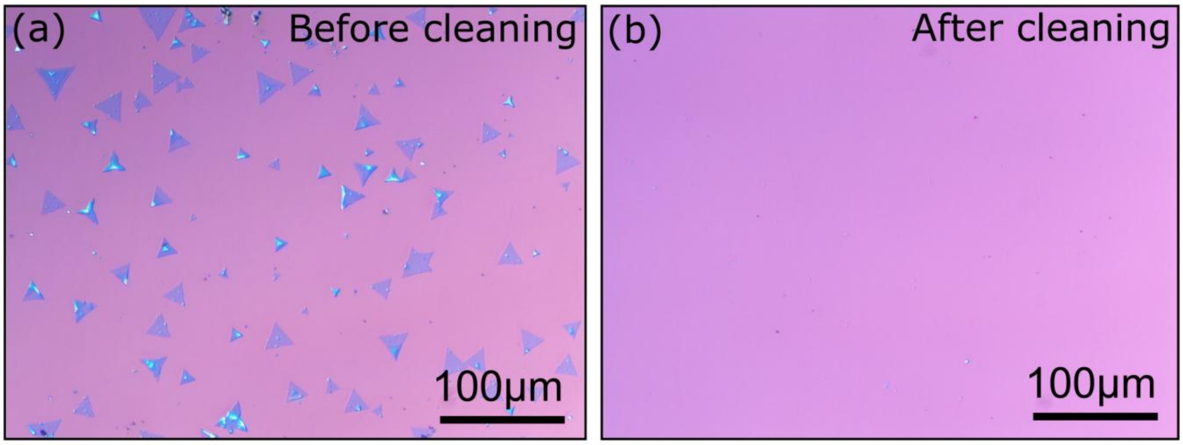

Figure S5: Macroscopic images of CVD-grown monolayer MoS_2 flakes before and after cleaning.

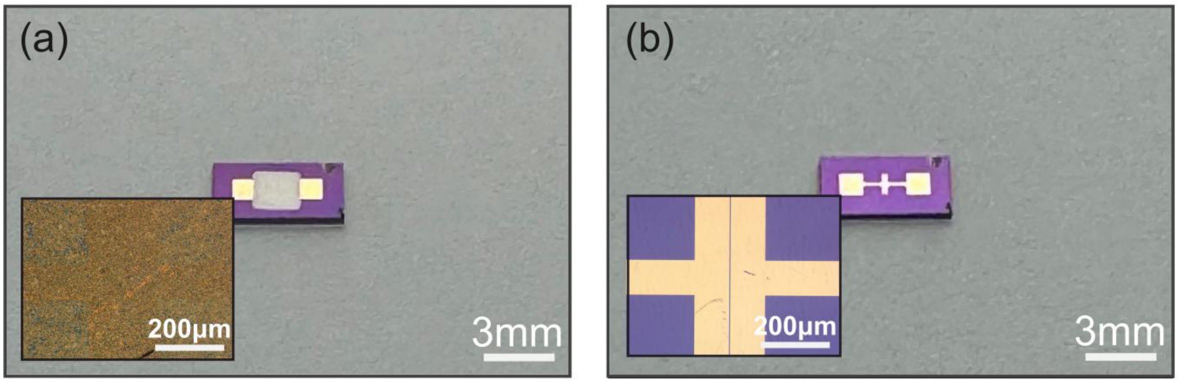

Figure S6: Macroscopic and optical microscopy images of the Ti/Au electrode-patterned SiO_2/Si substrate used for electrical characterization in the main text, shown before and after the cleaning process. The material used in this case is WSe_2.

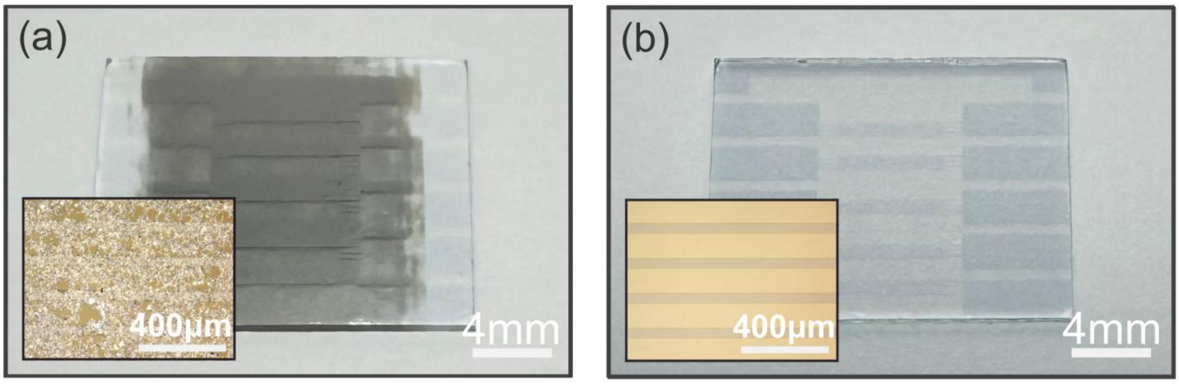

Figure S7: The macroscopic and optical microscopy images of indium tin oxide (ITO) substrates before and after cleaning. The case of MoS_2 is presented here as a representative example.

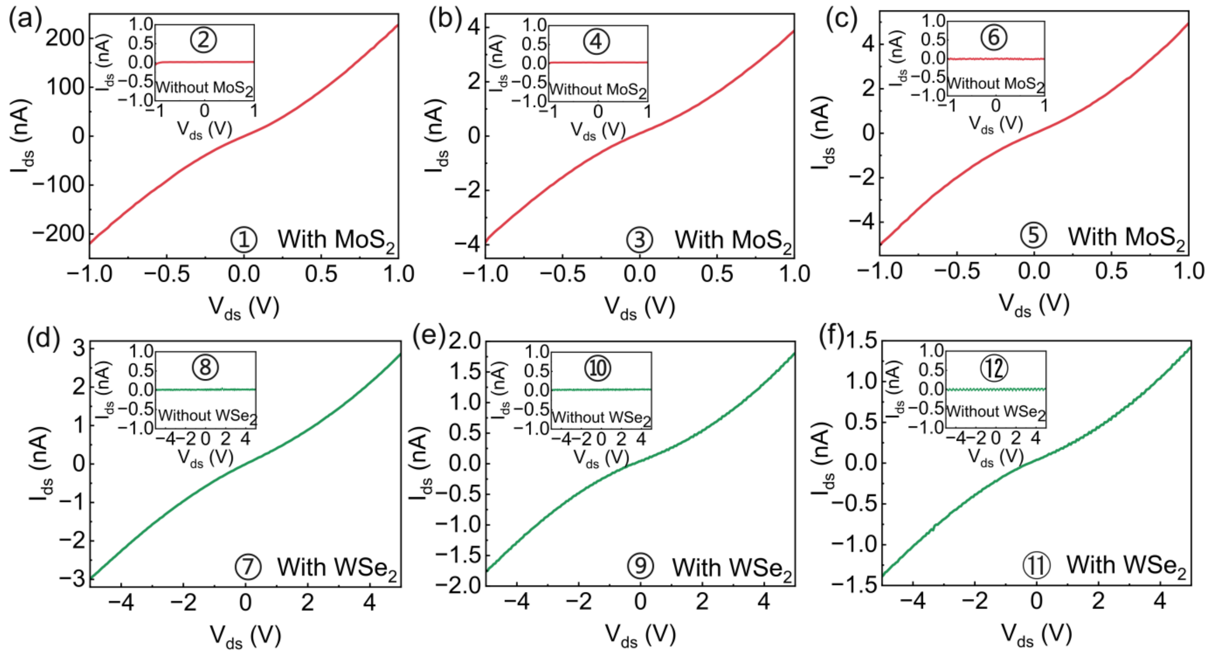

Figure S8: The output characteristics of repeatedly transferring MoS_2 and WSe_2 samples onto the same ITO substrate. (a-c) The main panels present the output characteristics of the MoS_2 device after the first, second, third transfer. (Insets) The output characteristics of the substrate after cleaning the device shown in (a-c), respectively. (d-f) The output characteristics of the WSe_2 device after the first, second, third transfer cycles, following three successful MoS_2 transfers. (Insets) The output characteristics of the cleaned substrate corresponding to the devices shown in (d-f), respectively.

To present the Raman mapping results as clearly as possible in the main manuscript, the integrated-intensity maps shown in Figure 4d-f were displayed as processed interpolated maps. The corresponding raw pixelated maps are provided here in Figure S9 to demonstrate that the observed contrast is not an artefact of interpolation. Both representations lead to the same conclusion, with the MoS_2-related Raman signal absent in the pristine electrode, clearly appearing after transfer, and returning to a near-background level after NMP cleaning.

The average Raman spectra shown in Figure 4g were obtained by averaging all spectra within each mapped region. The somewhat sharp or polygonal appearance of the MoS_2 peaks arises from the spectral sampling used in the mapping experiment. Because the spectra were acquired over a wide Raman-shift range with a limited number of spectral points, only a relatively small number of points fall within the MoS_2 Raman region. This affects the visual shape of the plotted peaks but does not affect the integrated-intensity analysis used here.

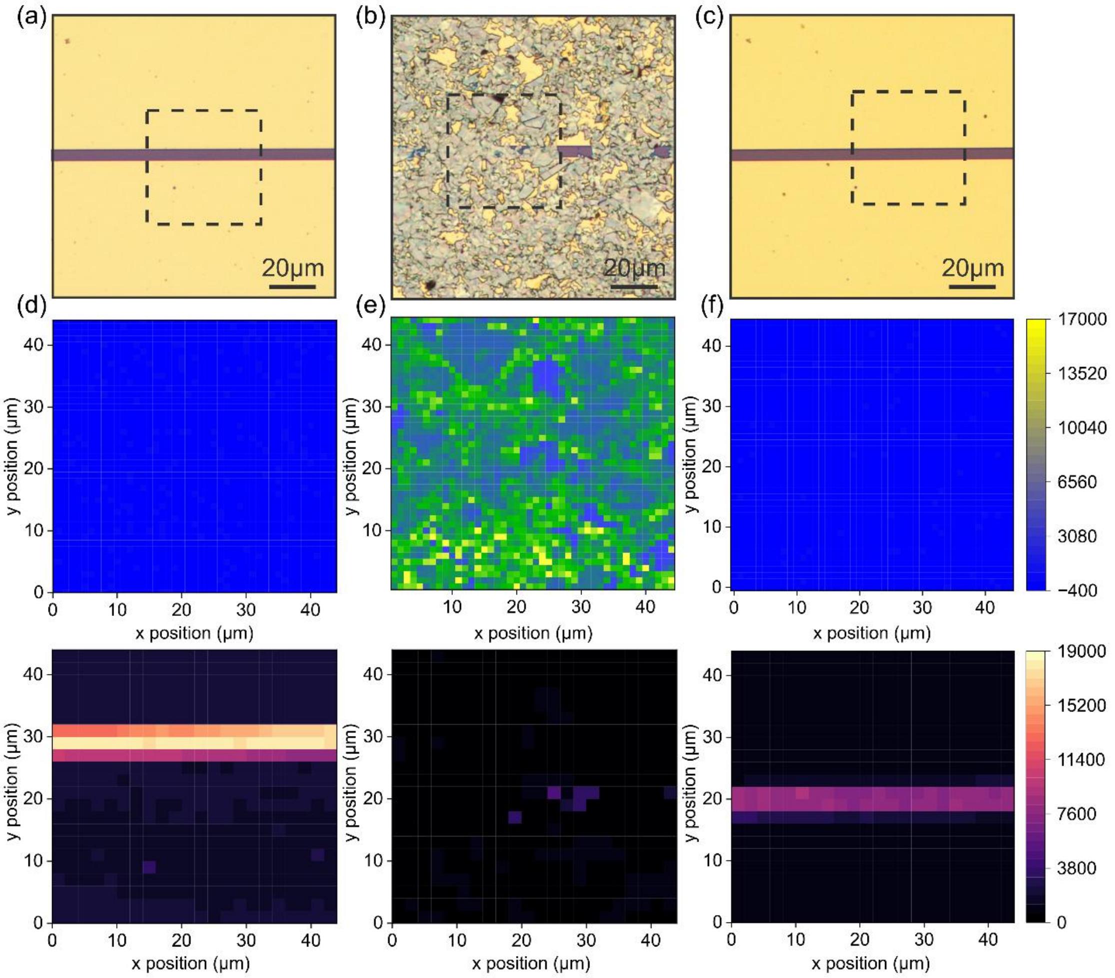

Figure S9: Raw pixelated Raman mapping analysis of the electrode surface in the pristine state, after roll-to-roll mechanically exfoliated MoS_2 transfer, and after NMP cleaning. (a–c) Optical microscopy images of the pristine state, after MoS_2 transfer, and after NMP cleaning, respectively. The dashed outlines indicate the Raman mapping areas. (d–f) Raw pixelated Raman integrated-intensity maps obtained by integrating the MoS_2 characteristic spectral window of 370-425 cm^{-1} after linear baseline correction. These maps correspond to the interpolated maps in Figure 4 and show the original pixel-level spatial distribution. (g–i) Raw pixelated Si 520 cm^{-1} Raman integrated-intensity maps of the same regions, used to distinguish the SiO_2/Si channel from the Au electrode region. The Si maps use a separate color scale from the MoS_2 maps because they represent a different Raman feature.

To further evaluate the effect of the transfer and cleaning process on the electrode surface, Kelvin probe force microscopy (KPFM) measurements were performed on the same electrode in two states, pristine electrode and after roll-to-roll mechanically exfoliated MoS_2 transfer followed by NMP cleaning. To distinguish between global and local surface changes, both large-area scans covering the electrode/channel region and smaller-area scans at higher magnification were recorded. The corresponding results are shown in Figures S10 and S11, respectively.

The large-area KPFM measurements shown in Figure S10 provide an overview of the electrode/channel region for the pristine electrode and after transfer followed by NMP cleaning. The topographic images do not reveal obvious large-scale residues or severe surface damage after processing. In the corresponding surface-potential (SP) maps, the cleaned electrode still exhibits a largely homogeneous potential distribution over the scanned region, without pronounced large-area electrostatic inhomogeneity. These results indicate that the transfer and NMP cleaning process does not introduce obvious large-scale disruption of the electrode surface.

To further examine the surface at higher spatial resolution, smaller-area KPFM scans were performed, as shown in Figure S11. The topographic images of the pristine and cleaned electrode appear broadly similar, with only minor local variations. The corresponding SP maps also remain largely homogeneous after cleaning. The SP histograms show that the distribution after transfer and cleaning is slightly broader than that of the pristine electrode, indicating that minor residual surface-state variations may remain after processing. These variations may arise from trace adsorbates, transfer-related residues, or subtle changes in the local surface environment. At the same time, the difference in the absolute mean SP value between the two measurements is not used for interpretation, since it can be affected by slight changes in the tip condition between scans. Therefore, the main conclusion from the KPFM measurements is that

the cleaning process substantially preserves the surface-potential uniformity of the electrode, even if exact recovery of the pristine distribution is not claimed. Overall, the KPFM results support the conclusion that NMP cleaning preserves a largely uniform electrode surface after roll-to-roll MoS_2 flake transfer, with no obvious large-area electrostatic inhomogeneity and only a modest broadening of the local SP distribution. Importantly, the KPFM analysis is not based on differences in the absolute mean SP value between the two measurements, since such differences can be influenced by changes in the KPFM tip condition between scans. Instead, the interpretation focuses on the spatial uniformity and width of the SP distribution. This interpretation is also consistent with the electrical characterization of MoS_2 and WSe_2 devices fabricated using recycled electrodes. Although transistor measurements do not directly quantify the electrode work function, the absence of a pronounced degradation or systematic shift in the device characteristics argues against a strong process-induced modification of the effective electrode work function or contact energetics. If the cleaning process had substantially altered the electrode electronic properties, noticeable changes in charge injection, contact resistance, or overall device characteristics would be expected, particularly in metal-contacted 2D semiconductor devices.

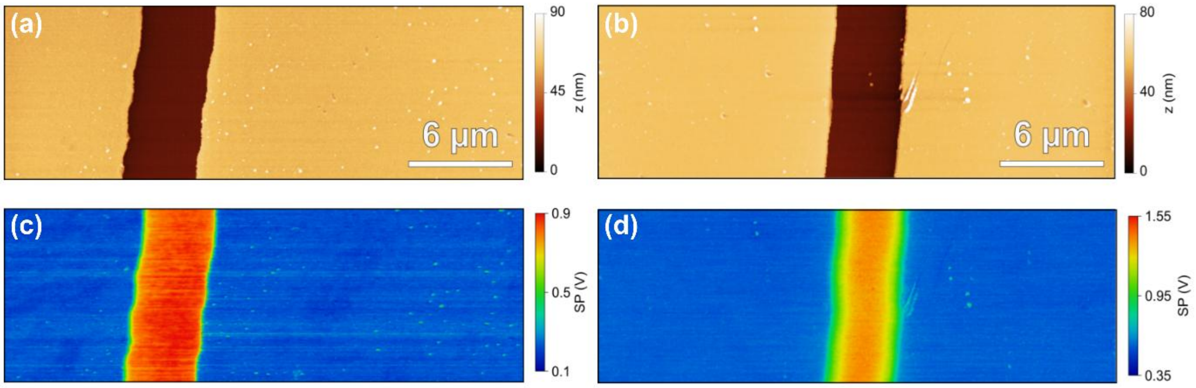

Figure S10: Large-area KPFM analysis of the pristine electrode and the electrode after roll-to-roll mechanically exfoliated MoS_2 transfer followed by NMP cleaning. (a,b) AFM topography images of the pristine electrode and the transfer-and-cleaned electrode, respectively. (c,d) Corresponding surface-potential (SP) maps of the same regions. The cleaned electrode retains a largely homogeneous SP distribution over the electrode/channel region, without pronounced large-area electrostatic inhomogeneity.

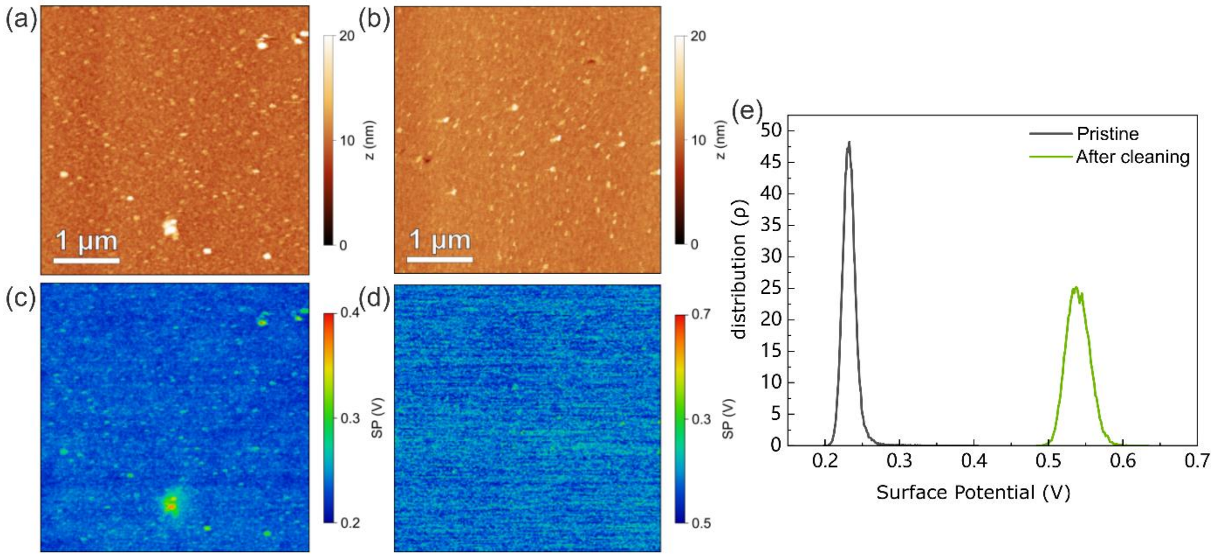

Figure S11: Higher-magnification KPFM analysis of the pristine electrode and the electrode after roll-to-roll mechanically exfoliated MoS_2 transfer followed by NMP cleaning. (a,b) AFM topography images of the pristine electrode and the transfer-and-cleaned electrode, respectively. (c,d) Corresponding surface-potential (SP) maps of the same regions. (e) Surface-potential histograms extracted from the maps.

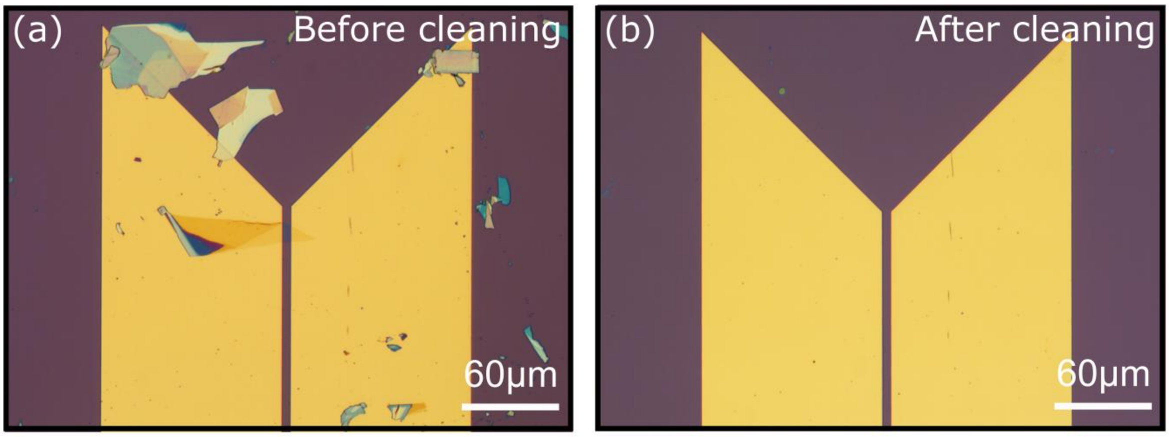

Figure S12: Macroscopic images of a monolayer MoS_2 flake transferred onto pre-patterned electrodes without annealing, before and after ultrasonic cleaning.

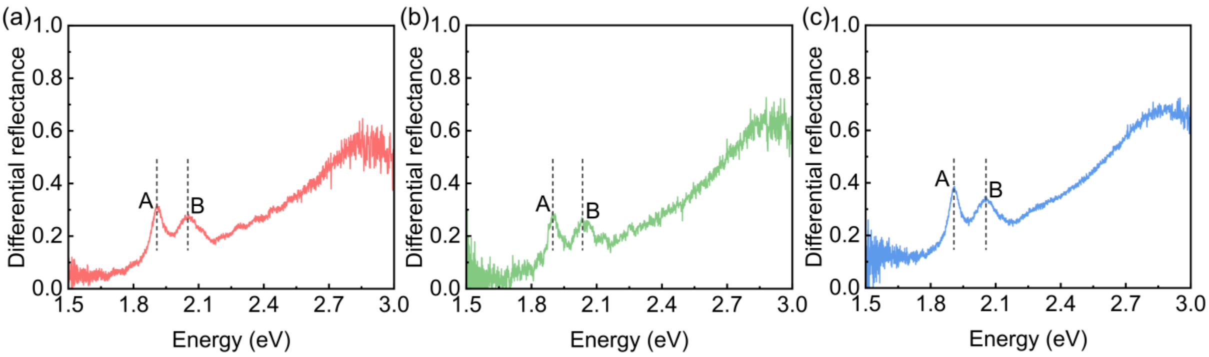

Figure S13: Differential reflectance spectra of monolayer MoS_2 flakes. Differential reflectance spectra of the monolayer MoS_2 flakes shown in Figures 5a, 5c, and S12 (panels a-c, respectively). All spectra exhibit the characteristic A and B exciton peaks, with the A exciton located around 1.90 eV.

Table S2: Field-effect mobility and ON/OFF ratio of monolayer MoS_2 FETs.

Devices	W_{eff} (μm)	g_m(20-40V) Forward (A/V)	g_m(20-40V) Backward (A/V)	μ_{FE} Forward (cm²/V·s)	μ_{FE} Backward (cm²/V·s)	ON/OFF Forward	ON/OFF Backward
Fig.5a	17.28	1.779×10^{-6}	1.858×10^{-7}	43.2	45.2	7.5×10^{6}	3.4×10^{6}
Fig.5c	8.37	4.112×10^{-7}	4.520×10^{-7}	22.0	22.7	8.5×10^{5}	1.1×10^{6}

Field-effect mobility (μ_{FE}) was extracted from the transfer characteristics in the linear regime according to

$$\mu_{FE} = \frac{L}{W} \cdot \frac{g_m}{C_{ox} V_{ds}}$$

$$g_m = \frac{dI_{ds}}{dV_g}$$

All devices were measured at V_{ds}=1 V using a SiO_2_back gate with oxide thickness t_{ox}= 290 nm. The gate-oxide capacitance per unit area was calculated as $C_{ox} = \varepsilon_0 \varepsilon_r / t_{ox}$=$1.19\times10^{-8}$ F/cm². The channel length is L= 5 μm. The effective channel width W_{eff} was determined from optical micrographs by measuring the MoS_2 flake overlap across the channel region at multiple positions along the channel and taking the harmonic mean. The transconductance g_m was obtained from a linear fit of the I_{ds} -V_g curve over V_g= 20-40 V using the high-current-range data. Forward and backward sweeps were analyzed separately, and a conservative single-value mobility was defined as the lower value between the two sweep directions.

The ON/OFF current ratio was calculated as $ON/OFF = I_{on}/I_{off}$ from the transfer curves. To ensure reliable values over the full dynamic range, stitched measurements

were used: I_{on} was taken from the high-current-range data, while I_{off} was taken from the low-current-range data. ON/OFF ratios were reported for both sweep directions, and a conservative single-value ON/OFF ratio was defined as the lower value between forward and backward sweeps.
